# 

**Published:** 1866-09

**Authors:** 


### CONTENTS.

ORIGINAL COMMUNICATIONS.

Chloroform and its administration........ 377
Address........ 380
Review........ 389
Stuffing Teeth........ 391
Make the Foundation firm........ 396

PROCEEDINGS OF SOCIETIES.

Minutes of the Ohio Dental College Association........ 399

SELECTIONS.

Rhigolene.—A Petroleum Naphtha for producing Anæsthesia by freezing........ 408
Aluminium as a base for Artificial Teeth........ 410
Ether vs. Chloroform........ 412
Another Chloroform case........ 413
Etherization........ 414
Chloroform........ 415
A Human Curiosity........ 415
Stomatoscope........ 416

EDITORIAL.

American Dental Association........ 416
An Address to the members of the Missouri Dental Association........ 419
A new Inhaler for all Anæsthetics........ 419
Pulp Extractors........ 420
Why Not?........ 421
New York College of Dentistry........ 422
Obituary—Dr. J. L. Nourse........ 376

